# How Does Attention Alter Length Perception? A Prism Adaptation Study

**DOI:** 10.3389/fpsyg.2020.02091

**Published:** 2020-08-18

**Authors:** Yong-Chun Cai, Xian Su, Yu-Mei Yang, Yu Pan, Lian Zhu, Li-Juan Luo

**Affiliations:** ^1^Department of Psychology and Behavioral Sciences, Zhejiang University, Hangzhou, China; ^2^Laboratory of Applied Brain and Cognitive Sciences, School of Business and Management, Shanghai International Studies University, Shanghai, China; ^3^School of Journalism and Communication, Shanghai International Studies University, Shanghai, China

**Keywords:** visual attention, length perception, appearance, prism adaptation, pseudoneglect

## Abstract

How perceived size (length) of an object is influenced by attention is in debate. Prism adaptation (PA), as a type of sensory motor adaptation, has been shown to affect performance on a variety of spatial tasks in both neglect patient and healthy individuals. It has been hypothesized that PA’s effects might be mediated by attentional mechanisms. In this study, we used PA to laterally shift spatial attention, and employed a precise psychophysical procedure to examine how the perceptual length of lines was influenced by this attentional shifting. Participants were presented with two separate lines in the left and right visual fields, and compared the length of the two lines. Forty-five healthy participants completed this line-length judgment task before and after a short period of adaptation to either left- (Experiment 1) or right-shifting (Experiment 2) prisms, or control goggles that did not shift the visual scene (Experiment 3). We found that participants initially tended to perceive the line presented in the left to be longer. This leftward bias of length perception was reduced by a short period of visuomotor adaptation to the left-deviating PA. However, for the right-shifting PA and plain glass goggles conditions, the initial length perception bias to the left line was unaffected. Mechanisms of this asymmetric effect of PA was discussed. Our results demonstrate that the length perception of a line can be influenced by a simple visuomotor adaptation, which might shift the spatial attention. This finding is consistent with the argument that attention can alter appearance.

## Introduction

Attention, as an essential process of human behavior, enables us to choose task relevant information from overwhelmed irrelevant stimulus (Carrasco, 2011). Numerous experiments have proved that attention can improve performance by decreasing reaction time or increasing response accuracy (Posner, 1980; Nakayama and Mackeben, 1989; Lu et al., 2012; Cai and Li, 2015). Some researchers have suggested that attention can affect subjective perceptual experience (Carrasco and Barbot, 2019). For example, Carrasco et al. reported that the subjective contrast of a grating was enhanced when spatial attention was shifted onto it (Carrasco et al., 2004; Liu et al., 2009). Other stimulus dimensions, such as perceptual size (Anton-Erxleben et al., 2007), spatial resolution (Gobell and Carrasco, 2005), motion speed (Turatto et al., 2007), motion coherence (Liu et al., 2006), perceptual organization (Barbot et al., 2018), and even face attractiveness (Stormer and Alvarez, 2016), have also been reported to be modulated by spatial attention. However, other researchers argued that the results supporting the attentional modulation on appearance were contaminated by decision/response biases and might not reflect a genuine change in subjective appearance (Prinzmetal et al., 2008; Schneider and Komlos, 2008). Zhou and colleagues recently found that how attention modulates subjective contrast depends on the physical contrast of stimuli: attention enhances the subjective contrast of low-contrast stimulus, whereas attenuates the subjective contrast of high-contrast stimulus (Zhou et al., 2018). This finding suggests that the direction of attentional effects on appearance may not be solidified but flexible according to the current task or stimulus property.

As the debate of contrast appearance, whether and how attention influences length perception is yet to be answered. There are three perspectives, that is, visual attention can attenuate, enhance and cannot alter length perception. Tsalet al. proposed that attention could attenuate length perception (Tsal and Shalev, 1996; Tsal et al., 2005). They used a hollow circle as attentional cue to manipulate attention, and presented a vertical line in left visual field, right visual field or central vision field. Subjects needed to judge which of lines that were previously shown was most similar to the length of the vertical line. Their result indicated that the attended line was perceived to be shorter than the unattended line (Tsal and Shalev, 1996; Tsal et al., 2005). In contrast, some other authors put forward that visual attention enhanced length perception. They suggested that when a line was attended, it appeared longer than the unattended line. Importantly, this enhancement of perceived line-length could be caused by both the exogenous attention (Toba et al., 2011) and endogenous attention (Masin, 2003). Others, however, insisted that attention could not alter perceived length. For example, Prinzmetal and Wilson (1997) required subjects to estimate the length of a line while completing a second concurrent task (letter identification). By using this dual-task paradigm to manipulate attention, they found that the primary effect of attention was to reduce the variability of line length adjustments, but not to affect the subjective length of a line (Prinzmetal and Wilson, 1997).

PA is a type of sensory motor adaptation that affects performance on a variety of spatial tasks in both neglect patient (Rossetti et al., 1998; Pisella et al., 2002; Redding and Wallace, 2006; Jacquin-Courtois et al., 2013) and healthy individuals (Colent et al., 2000; Michel et al., 2003; Loftus et al., 2009; Schintu et al., 2014). It is suggested that PA might exert its effects on various spatial tasks through shifting spatial attention to the spatial space contralateral to the adaptation direction (Crottaz-Herbette et al., 2014; Martin-Arevalo et al., 2016). We wondered how this type of attention allocation influences length perception. Previous studies reported that leftward PA in healthy individuals induces neglect-like biases in visuospatial tasks (Colent et al., 2000; Michel et al., 2003; Loftus et al., 2009). For example, after adaptation to leftward-deviating prism, subjects tended to judge the midpoint of a line shifted toward right side of the true center in the line bisection task (Schintu et al., 2014; McIntosh et al., 2019). However, in these studies, subjects were required to either mark the midpoint of a line manually or judge whether a pre-marked line is correctly bisected. It is possible that the effects of PA on line bisection did not result from the modulation of PA on length perception but was due to the perceptual error of the transector position.

Indeed, a recent study reported that there is a weak, if any, correlation between the performances of the line bisection task and a length matching (or comparing) task, in which participants were required to compare the horizontal length of two rectangles and judge which of them was longer (McIntosh et al., 2017). This finding confirms the above speculation that effects of PA on the line bisection do not necessarily result from the effects of PA on length perception. In this study, we attempted to examine whether and how prism adaptation influences length perception with the length comparing task and psychophysical procedures that could precisely tap the apparent length of lines. Subjects were presented with two separate lines in the left and right visual fields, and were required to compare the length of the two lines. This procedure is similar to previous studies investigating how attention alters subjective perception of other visual dimensions (Carrasco et al., 2004; Gobell and Carrasco, 2005; Liu et al., 2006, 2009; Anton-Erxleben et al., 2007; Turatto et al., 2007; Stormer and Alvarez, 2016; Kirsch et al., 2018; Zhou et al., 2018).

## Materials and Methods

### Participants

In total, 45 naive observers, all from Zhejiang University, with normal or corrected to normal vision participated in this study. Fifteen participants (9 males; mean age = 19.1, *SD* = 1.67 years) underwent adaptation to leftward-deviating prisms (Experiment 1). Fifteen participants (13 males; mean age = 19.3, *SD* = 1.52 years) were adapted using rightward-deviating prisms (Experiment 2). Fifteen participants (8 males; mean age = 18.9, *SD* = 1.33 years) completed Experiment 3 (plain glasses condition). All participants gave informed consent and were either paid or compensated with course credits for their participation. Written informed consent was obtained from the individual for the publication of any potentially identifiable images or data included in this article. The Research Ethics Board of Zhejiang University approved all the experimental procedures.

### Apparatus, Stimuli, and Procedure

All stimuli were generated using Matlab (MathWorks, Natick, MA) with Psychophysics Toolbox (Brainard, 1997; Pelli, 1997) and all experiments were conducted in a dimly lit room.

**FIGURE 1:**
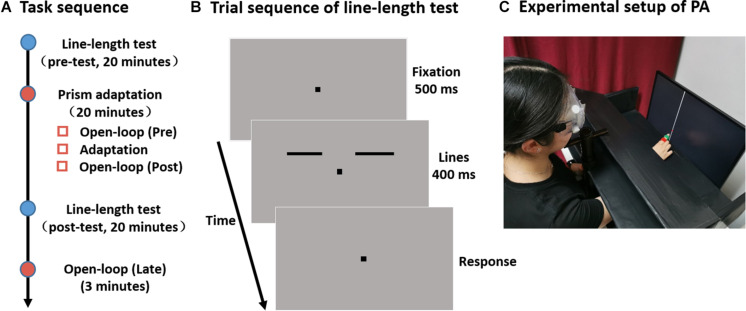
Experimental design. **(A)** Experimental timeline. **(B)** Sequence of events in a single trial in the line-length test. Two separate horizontal lines are presented to the left and right side of the fixation point. The length of one line (the reference line) is fixed at 4°, while the length of the other line (the test line) is varied. Subjects are required to judge which side of the line is longer. **(C)** Participants were comfortably seated with their head positioned on a chinrest in front of a screen. They were asked to point with their right index finger to a vertical white line. To measure their pointing position on the screen, a laser pointer, which could turn on and off remotely by the experimenter, was fixed on the right index finger of participants. Participants could see (in sensorimotor adaptation session, as shown here) or could not see (in open-loop pointing session, not shown) their pointing movement and the laser point on the screen.

Each experiment was divided into four main parts: a line-length test session performed once before (pre-) and once after adaptation (post-), the adaptation procedure, and an open-loop measurement following the post-line-length test (Figure 1A).

### Line-Length Test

For the line-length test, stimuli were displayed on a CRT monitor (21” Dell UltraScan P1130; 1024 × 768 resolution; 100-Hz refresh rate). The viewing distance was 74 cm from the screen with the head stabilized by a chinrest. Stimuli were all black (0 cd/m^2^) drawn against a gray (38 cd/m^2^) background. A small fixation square (0.2° × 0.2°) was presented on the center of the screen throughout each trial. Two horizontal lines (0.12° thick) were situated 2° above the fixation point with one on the left and the other on the right. The inner endpoint (i.e., the endpoint near to the fixation) of each line was at an eccentricity of 2° along the horizontal meridian. One of the line was the *reference line* and its length was fixed at 4°. The other line was the *test line* and its length varied in 11 log increments following a one-up-one-down staircase rule. The test line was randomly presented to one side of the fixation, and the reference line was presented to the opposite side.

A schematic of a trial sequence is shown in Figure 1B. After the presentation of the fixation point (500 ms), a pair of lines was presented for 400 ms. Participants were requested to judge which side of the line was longer by pressing the appropriate keys on the keyboard (“f” for left and “j” for right with their left and right hand, respectively). Each line-length test session took about 20 min and consisted three 80-trial blocks. For each participant, the percentage of “test-line longer” judgments was plotted as a function of the length of test line and these data were then fitted to a cumulative Gaussian function. The length of the test line at which the “test-line longer” responses reach 50% of the time was taken as the point of subjective equality (PSE).

### Prism Adaptation

Participants were comfortably seated with their head positioned on a chinrest at a 60 cm distance in front of a screen (Samsung LS24E390HL; 1920 × 1080 resolution; 60 Hz refresh rate) and completed three sessions of open-loop pointing task and one session sensorimotor adaptation task (Figure 1A). The setups for the line-length test and for the prism adaptation were in the same room, and participants immediately moved to the next session when one session was completed. The experimental setup for prism adaptation is demonstrated in Figure 1C.

During each open-loop pointing session, participants were asked to point with their right index finger to a vertical white line (0.13° thick) of a length equivalent to the screen height. The line was possibly positioned at 0°, −3°, or 3° horizontally from their body midline. To measure their pointing position on the screen, a laser pointer, that could turn on and off remotely by the experimenter, was fixed on the right index finger of participant. The laser pointer kept turning off during each pointing movement. In each trial, participants informed the experimenter by voice when their pointing movement finished, and their fingers kept at the pointing direction. Thereafter, the experimenter turned on the laser pointer remotely and clicked the laser point projected on the screen using a mouse to record each pointing position. A board was placed above the participants’ hands so that they could not see either their pointing movement or the laser point on the screen. Prior to each pointing, participants placed their right hand at the starting position, just in front of their chest. When the line was presented, they executed a one-shot movement at a fast but comfortable speed, and returned their hand to the starting position when instructed by the experimenter. Each participant made 12 pointing movements, and the open-loop pointing measure was the average of these 12 pointing movements.

During adaptation, participants were fitted with prism goggles which deviated their visual field by 14° either leftward (Experiment 1) or rightward (Experiment 2), or in a control group, with plain glass goggles (Experiment 3). They performed 50 pointing movements toward a vertical line positioned at either 0°, −10°, or 10° horizontally from their body midline. They could not see their hand when it was in the starting position or during the first third of the pointing movement, but could see the rest of the pointing movement and the laser point projected on the screen. Participants were instructed to make a ballistic movement and to correct any errors on the subsequent movement. The goggles were removed and participants were tested in the open-loop pointing once again directly after adaptation as well as at the end of the experiment (i.e., 20 min after adaptation), to assess whether the sensorimotor aftereffects were still present (Figure 1A).

## Results

### Experiment 1: Leftward-Deviating PA

#### Open-Loop Pointing

**FIGURE 2:**
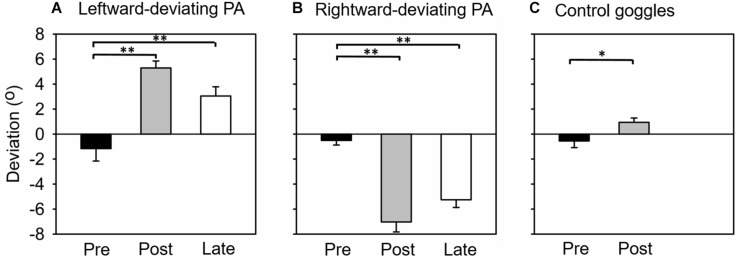
Mean open-loop pointing error (degree) in each experiment. **(A)** Experiment 1, adaptation to leftward-deviating prism. **(B)** Experiment 2, adaptation to rightward-deviating prism. **(C)** Experiment 3, control group (wearing plain glass goggles). Pre, Post, and Late mean that the open-loop pointing tested before adaptation, immediately after adaptation, and at the end of the experiment, respectively. Error bars indicate one standard error. *indicates *p* < 0.05, and **p < 0.05.

The difference between pointing positions before and after PA was used to assess whether participants adapted to prisms and whether they remained adapted during the line-length test. Figure 2A shows the average pointing error for the three open-loop pointing task sessions. A one-way repeated-measures ANOVA revealed a significant effect of session [*F*(2, 28) = 39.22, *p* < 0.001]. In the pre-PA session, participants’ average pointing position slightly deviated toward the left of the target (pointing error, Mean ± SEM = −1.16° ± 0.99°), while in the post-PA session the pointing error reversed and deviated to the right (pointing error, Mean ± SEM = 5.29° ± 0.56°). In the final session of the open-loop task (i.e., late open-loop session, Figure 1A), pointing position still deviated to the right of the target (pointing error, Mean ± SEM = 3.05° ± 0.73°). *Post-hoc* tests revealed that open-loop pointing at the pre-PA session differed significantly from each of the two post-adaptation measurements (*ps* < 0.01, Bonferroni corrected). This result indicated that leftward-deviating PA generated a significant rightward sensorimotor aftereffect, which could last until the completion of the experiment.

#### Line-Length Test

**FIGURE 3:**
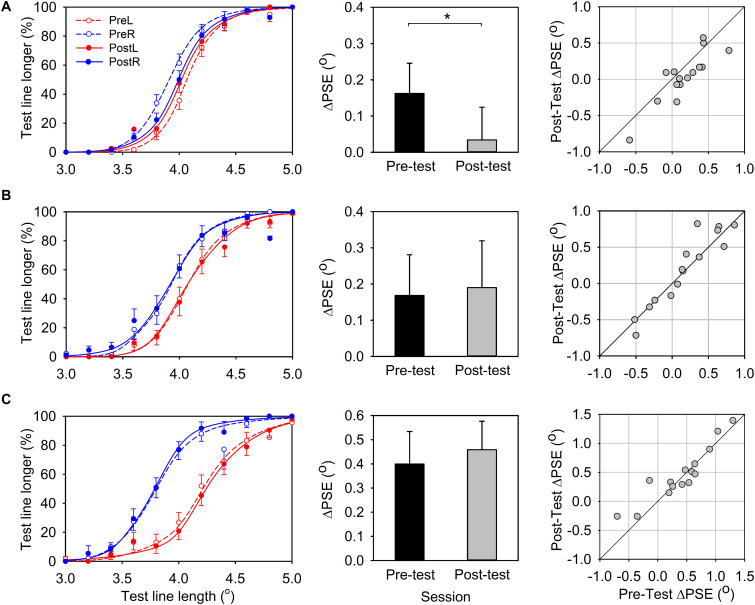
Results for line-length comparative task. **(A)** Experiment 1, leftward-deviating PA. **(B)** Experiment 2, rightward-deviating PA. **(C)** Experiment 3, control group. **Left panels:** Percentage of “test line longer” response is plotted as a function of the test line’s physical length. Solid and dashed lines represent pre- and post-PA condition, respectively, and red and green lines represent the reference line located in the left and right visual field, respectively. **Center panels:** The PSE difference (ΔPSE) between the conditions of reference line in the left and in the right visual field (black bars, pre-PA condition; gray bars, post-PA condition). **Right panels:**ΔPSEs for the pre-PA condition are plotted vs. ΔPSEs for the post-PA condition. Error bars indicate one standard error. *indicates *p* < 0.05, and **p < 0.05.

The average percentage of “test line longer” responses was plotted as a function of the length of the test line and the mean psychometric function was obtained by averaging the fitted psychometric function of each participant (Figure 3A, left panel). The test-line length at which the response function reaches 50% is PSE, namely, the apparent length for the reference line.

In the pre-test session, when the reference line was positioned in the left visual field, the response function shifted to the right and the PSE (Mean ± SEM = 4.08° ± 0.04°) was larger than 4^*o*^, which was the physical size of the reference line. This indicated that the test line in the right must be longer than 4^*o*^ so that its length appeared the same as the reference line in the left (Figure 3A, left panel, the red dashed line). In contrast, when the reference line was presented in the right visual field, the response function shifted to the left and the PSE (Mean ± SEM = 3.93° ± 0.04°) was smaller than 4^*o*^. This indicated that the test line in the left must be shorter than 4^*o*^ so that it appeared the same length as the reference line (Figure 3A, left panel, the blue dashed line). These results together suggested that a line presented to the left visual field would appear longer than presented to the right visual field. This is consistent with the leftward bias of healthy participants observed in previous studies (i.e., pseudoneglect phenomenon). We calculated the PSE difference between the conditions of reference in the left and in the right visual field (ΔPSE = PSE_left_ - PSE_right_) to evaluate this leftward bias effect. A positive ΔPSE would indicate a leftward bias and a negative ΔPSE would indicate a rightward bias. TheΔPSE was marginally significantly different from zero [ΔPSE, Mean ± SEM = 0.16° ± 0.08°, *t*(14) = 1.95, *p* = 0.07], reaffirming the leftward bias for the line length judgment task in the pre-test session.

In the post-test session, the lateral shift of the two response functions was smaller than that in the pre-test session, and the PSEs in the two conditions (reference-line in the right, Mean ± SEM = 4.00° ± 0.05°; reference-line in the left, Mean ± SEM = 4.03° ± 0.04°) were close to 4° (Figure 3A, left panel, solid lines). TheΔPSE was not significantly different from zero [ΔPSE, Mean ± SEM = 0.03° ± 0.09°, *t*(14) = 0.38, *p* = 0.71], suggesting there was no significant spatial bias in the post-test line length judgment. More importantly, the ΔPSE of the post-test session was significantly smaller than that in the pre-test session [Figure 3A, center panel; paired-test, *t*(14) = −2.85, *p* = 0.01]. This average pattern was also evident for individual data (Figure 3A, left panel).

In summary, the results of Experiment 1 showed that healthy participants demonstrated leftward bias in the line-length judgment task in the pre-test session, whereas after leftward-deviating PA this leftward bias was eliminated.

### Experiment 2: Rightward-Deviating PA

In this experiment, we aimed to test how rightward-deviating PA affected the line-length judgment. Participants underwent the same procedures as in Experiment 1, with the only difference that they wore rightward-deviating prism goggles in the PA session.

#### Open-Loop Pointing

Figure 2B shows the average pointing error for the three open-loop pointing task sessions. A one-way repeated-measures ANOVA revealed a significant effect of session [*F*(2, 28) = 74.59, *p* < 0.001]. In the pre-PA session, participants’ average pointing position slightly deviated toward the left of the target (pointing error, Mean ± SEM = −0.51° ± 0.37°). In the post-PA session, the leftward pointing error was obviously increased (pointing error, Mean ± SEM = −7.03° ± 0.80°), and this enlarged leftward pointing error was sustained in the late open-loop session (pointing error, Mean ± SEM = −5.25° ± 0.62°). *Post-hoc* tests revealed that open-loop pointing at the pre-PA session differed significantly from each of the two post-adaptation measurements (*p*s < 0.01, Bonferroni corrected). This result indicated that rightward-deviating PA generated a significant leftward sensorimotor aftereffect, which could last until the completion of the experiment.

#### Line-Length Test

Figure 3B shows the results of line-length test. In the pre-test session, similar to Experiment 1 (leftward-deviating PA), the average PSE for the reference-line in the left visual field condition (Mean ± SEM = 4.09° ± 0.06°) and the reference-line in the right visual field condition (Mean ± SEM = 3.93° ± 0.06°) was shifted to the right and left of 4^*o*^ point, respectively (Figure 3B, left panel). TheΔPSE (Mean ± SEM = 0.17° ± 0.11°) was larger than zero, suggesting the leftward bias for the line-length test. In the post-test session, the average PSEs for the two conditions (PSE for reference-line in the left, Mean ± SEM = 4.10° ± 0.06°; PSE for reference-line in the right, Mean ± SEM = 3.91° ± 0.07°) were similar to that in the pre-test session. TheΔPSE (Mean ± SEM = 0.19° ± 0.13°) was not significantly different from that in the pre-test session [*t*(14) = 0.48, *p* = 0.64; Figure 3B, center panel]. This is in contrast to the result of leftward-deviating PA group (Experiment 1) and indicates that rightward-deviating PA cannot influence the line length perception. The individual data also supports this conclusion (Figure 3B, right panel).

### Experiment 3: Sham PA

In this experiment, we aimed to exam whether experimental procedure *per se* of Experiments 1 and 2 would affect the line perception. Therefore, participants wore plain glass goggles to complete the open-loop pointing task and adaptation task. Other procedures were the same as that in Experiments 1 and 2.

#### Open-Loop Pointing

In this experiment, participants only completed pre- and post-PA open-loop pointing sessions. Figure 2C shows the average pointing error for the two sessions. Unexpectedly, a one-way repeated-measures ANOVA revealed significant effect of session [*F*(1, 14) = 8.55, *p* < 0.05]. Participants’ average pointing error was −0.55° ± 0.54° and 0.94° ± 0.35° in the pre-PA and post-PA session, respectively. This result indicated that, in this experiment, sham PA induced a slight rightward sensorimotor aftereffect.

#### Line-Length Test

Figure 3C shows the results of line-length test. In the pre-test session, the average PSEs for conditions of the reference-line in the left visual field (Mean ± SEM = 4.22° ± 0.07°) and the reference-line in the right visual field (Mean ± SEM = 3.82° ± 0.06°) were shifted to the right and left of 4^*o*^ point, respectively (Figure 3C, left panel). TheΔPSE (Mean ± SEM = 0.40° ± 0.13°) was larger than zero, showing the leftward bias for the line-length test. In the post-test session, the average PSEs for the two conditions (PSE for reference-line in the left, Mean ± SEM = 4.25° ± 0.07°; PSE for reference-line in the right, Mean ± SEM = 3.79° ± 0.05°) were similar to that in the pre-test session. The ΔPSE (Mean ± SEM = 0.46° ± 0.12°) was not different from that in the pre-test session [*t*(14) = 1.14, *p* = 0.27; Figure 3C, center panel]. This result indicates that sham PA cannot influence the line length perception. The individual data also supports this conclusion (Figure 3C, right panel).

## Comparing the Results of the Three Experiments

To further confirm that different direction of prism would produce different PA effects on length perception, we performed a 3^∗^2 ANOVA for the ΔPSE between Experiments 1–3 with adaptation condition (Exp. 1, left-deviating PA; Exp. 2, right-deviating PA; and Exp. 3, sham PA) as a between-subjects factor and test-session (pre-test vs. post-test) as a within-subjects factor. The main effects of the two factors were not significant [adaptation condition: *F*(2, 42) = 2.44, *p* = 0.1; test-session: *F*(1, 42) = 0.33, *p* = 0.57]. The interaction between the two factors was significant [*F*(2, 42) = 4.39, *p* = 0.02], indicating that the PA effect varied with the adaptation condition. *Post-hoc* tests comparing pre-test and post-test revealed that significant adaptation effect was only observed in the left-deviating condition [i.e., Experiment 1, *F*(1, 14) = 8.12, *p* = 0.01], but neither the right-deviating condition [i.e., Experiment 2, *F*(1, 14) = 0.24, *p* = 0.64] nor the sham PA condition [i.e., Experiment 3, *F*(1, 14) = 1.30, *p* = 0.27]. This analysis confirmed that only left-deviating prim reduces the left hemispace bias in the line-length perception.

## Discussion

The present study investigated the influence of PA on length perception in healthy individuals. Participants compared subjective length of two separate lines, presented in the left and right visual fields, before and after left-deviating PA, right-deviating PA or wearing control goggles fitted with plain glass lenses. Results showed that participants initially tended to judge the length of the line on the left side to be longer than that on the right side, demonstrating the leftward pseudoneglect bias (Bowers and Heilman, 1980; Jewell and McCourt, 2000). This leftward bias was reduced by left-deviating PA, but was unaffected in the right-deviating PA condition and wearing the plain glass goggles.

Many studies have reported that leftward prism adaptation could reduce or even reverse the leftward bias (“pseudoneglect”) in the line bisection task for heathy subjects (Colent et al., 2000; Michel et al., 2003; Schintu et al., 2014). In these studies, subjects were presented with a continuous line and were required to either mark the midpoint of a line manually (i.e., the manual line bisection task) or judge whether a pre-marked line is correctly bisected (i.e., the landmark task) (McIntosh et al., 2019). These results might provide evidence for the influence of PA on length perception. However, because these studies used continuous lines as stimuli, the comparison of the length between the left and right parts of a line could be affected by the perceptual position of the transector on the line. Therefore, the effects of PA on line bisection might not result from the modulation of PA on length perception but be ascribed to the perceptual error of the transector position. Consistent with this argument, a recent study reported that there is no correlation between the performances of the manual line bisection task and the length matching (or comparing) task, and only a weak correlation between the performances of the landmark task and the length matching task (McIntosh et al., 2017). We were first to require subjects to compare the length of two separate lines and evaluated how the perceived length of lines was impacted by the sensorimotor adaptation. This procedure was wildly adopted in previous studies to investigate how attention alters the appearance of contrast, spatial frequency, motion speed, and size (Carrasco et al., 2004; Gobell and Carrasco, 2005; Liu et al., 2006; Anton-Erxleben et al., 2007; Turatto et al., 2007; Liu et al., 2009; Kirsch et al., 2018; Zhou et al., 2018). We replicated the leftward pseudoneglect bias for the line-length perception in the pre-PA session and found that the left-deviating PA reduced this leftward bias in the post-PA session. Our results suggest that PA can really exert effects on the length perception.

Previous studies have reported that only leftward-deviating prisms induces a rightward shift both in the manual line bisection task and in the landmark task, while rightward-deviating prisms seem not to have the converse effect (Colent et al., 2000; Schintu et al., 2014, 2017). Similarly, in our line-length comparative task, we also found that left-shifting PA could impair the leftward bias in length perception, whereas right-shifting PA had no effect. The aftereffect of prism adaptation may be linked to either low-level sensorimotor plasticity or high-level cognitive processes (Redding and Wallace, 2006, 2010). Given that prism adaptation in healthy subjects produces symmetric sensorimotor aftereffects (Welch et al., 1993; Redding et al., 2005; Schintu et al., 2017), the asymmetric influence of PA on length perception in the present study is not likely attributable to the low-level sensorimotor aftereffects, but may be due to the alteration of high-level cognitive processing such as spatial representation. The bias of visuospatial representation is assumed to be related to the balance of competition between the two brain hemispheres (Kinsbourne, 1977). It has been hypothesized that PA exerts effects on visuospatial tasks through modulating the activity of the contralateral posterior parietal cortex (PPC), which in turn alters the interhemispheric equilibrium (Pisella et al., 2006; Striemer and Danckert, 2010; Newport and Schenk, 2012). Because of the asymmetries in interhemispheric inhibition between the left and right parietal cortex (Koch et al., 2011), leftward- and rightward-deviating PA may affect their contralateral parietal cortex differently thereby causing different effects on spatial tasks. In accordance with this explanation, many other cognitive processing involving spatial representation, such as mental number/alphabetic line bisection (Loftus et al., 2008; Nicholls et al., 2008), auditory perception (Michel et al., 2019), and time representation (Anelli and Frassinetti, 2019), is also modulated by leftward- and rightward-deviating PA in different ways.

An alternative explanation is that leftward-deviating PA biases spatial attention allocation to the right hemispace and thus leads to the length of the line in the right side appearing longer than that in the left side. Indeed, many studies have reported that PA can asymmetrically alter the orienting of spatial attention (Striemer et al., 2006; Striemer and Danckert, 2007; Clarke and Crottaz-Herbette, 2016; Martin-Arevalo et al., 2016). For example, Martín-Arevalo and colleagues exploited the event-related potentials to test how PA affected electrophysiological markers of attentional processes in the healthy human brain (Martin-Arevalo et al., 2016). They found that left-shifting PA affected early stage electrophysiological components (the cue-locked N1 and the target-locked P1), that are known to be related to attentional processes, whereas right-shifting PA had no effect on these components. These null results of right-shifting PA might be explained by the right parietal dominance for spatial attention (Corbetta and Shulman, 2002; Thiebaut de Schotten et al., 2011). As mentioned above, PA may exert its effect through inhibiting the activity of the contralateral PPC, a critical region mediating spatial attention (Pisella et al., 2006; Striemer and Danckert, 2010; Newport and Schenk, 2012). The left-shifting PA would interfere the function of right parietal cortex and in turn impair the attentional performance in the left hemispace, while the interference of the right-shifting PA on the left parietal cortex would not manifest any attentional effect. This assumption is consistent with the finding that both TMS and tDCS modulate attentional tasks in the contralateral hemispace only when the stimulation was applied over the right PPC (Chambers et al., 2004; Roy et al., 2015).

If the effects of PA were mediated by redistribution of spatial attention, PA should influence the perceptual length in a manner similar to spatial cueing. Using the same length/size comparative task as the present study, previous studies found that the perceived length or size of a target would be increased when spatial attention was pre-directed to the position of the target (Masin, 2003; Anton-Erxleben et al., 2007; Toba et al., 2011; Kirsch et al., 2018). Given the PA effects on spatial attention and the attentional effects on length perception, it is possible that leftward-deviating PA shifted spatial attention to the right visual field, which in turn amplified the perceived length of the line in the right visual field.

A number of studies have revealed that attention alters the appearance of many static and dynamic basic visual dimensions (for review, see Carrasco and Barbot, 2019). If PA modulates cognitive processing through allocation of spatial attention, it should not only influence spatial related task (e.g., length comparative task, line bisection task, mental number line bisection task), but also exert effects on none-spatial related perception, such as luminance, contrast, spatial frequency, and so on. Only few studies have attempted to address the question. For example, Loftus and colleagues observed that a forced choice judgment between two mirror-reversed luminance gradients (grayscales task) was influenced by prism adaptation (Loftus et al., 2009). However, their results may not reflect pure influences of PA on perceived luminance, but be contaminated by spatial factors, as in their experiment, each grayscale spread spatially with one end in the left and the other in the right visual field. In future studies, it will be important to address whether PA alters the appearance of none-spatial visual features.

It is worth to note that the sham PA induced a slight rightward sensorimotor aftereffect (Experiment 3, Figure 2C). The effect was small but significant. This unexpected sensorimotor aftereffect in the sham PA condition has also been report by other authors (e.g., Martin-Arevalo et al., 2016), and could be due to participants correcting their natural leftward kinematic errors (i.e., pseudoneglect) during the pointing procedure.

In conclusion, the current results demonstrate that PA affects length perception in healthy subjects. The leftward-deviating prism adaptation reduces leftward perceptual pseudoneglect bias in the task of comparing two lines presented laterally in the left and right hemispaces, whereas the right-shifting prism adaptation has no effect. This asymmetry implies that our results are due to high-level cognitive disturbance induced by prism adaptation.

## Data Availability Statement

The raw data supporting the conclusions of this article will be made available by the authors, without undue reservation.

## Ethics Statement

The studies involving human participants were reviewed and approved by the Research Ethics Board of Zhejiang University. The patients/participants provided their written informed consent to participate in this study.

## Author Contributions

Y-CC, Y-MY, and L-JL designed the experiments. Y-CC, YM-Y, and XS conducted the experiments, analyzed the data, and interpreted the results. Y-CC, L-JL, YP, and LZ wrote the manuscript. All authors contributed to the article and approved the submitted version.

## Conflict of Interest

The authors declare that the research was conducted in the absence of any commercial or financial relationships that could be construed as a potential conflict of interest.
